# Efficacy and safety of external therapy of TCM for diabetic peripheral vascular disease: A protocol for systematic review and meta-analysis

**DOI:** 10.1097/MD.0000000000032362

**Published:** 2022-12-23

**Authors:** Tingwei Ding, Lei Sheng, Haiyu Zhu, Hongyi Guan, Ying Wang, Haoze Guo, Yunpeng Bi, Bailin Song

**Affiliations:** a Department of Acupuncture and Tuina, Changchun University of Chinese Medicine, Changchun, China; b Department of Chinese Medicine, Changchun University of Chinese Medicine, Changchun, China.

**Keywords:** diabetic peripheral vascular disease, external therapy, protocol, systematic review, traditional Chinese medicine

## Abstract

In modern society, the incidence of diabetes is increasing yearly, and poor glycemic control can accelerate the progression and severity of diabetic peripheral vascular disease. External treatment with traditional Chinese medicine (TCM) has been widely used in the treatment of diabetes, but there is no systematic review on the external treatment of TCM for diabetic peripheral vascular disease; therefore, this study aimed to conduct a meta-analysis on the clinical efficacy and safety of external treatment of TCM in the treatment of diabetic peripheral vascular diseases.

**Methods:**

We searched The Cochrane Library, PubMed, Web of Science, EMBASE, China Science and Technology Journal, China National Knowledge Infrastructure, Wanfang, Chinese Biomedical Literature Database, and other sources from their respective inception dates to October 20, 2022, to identify potentially eligible studies. We will use Review Manager 5.4 software provided by the Cochrane Collaborative Network for statistical analysis. We then assessed the quality and risk of the included studies and observed their outcome measures.

**Results:**

Changes in the bilateral femoral artery, popliteal artery, dorsal artery diameter, blood flow, ankle/brachial index, and dorsal foot temperature: An overall reduction in symptoms (a reduction in diabetes-related symptoms that occur only during the intervention or as a result of the intervention, including peripheral vascular disease).

**Conclusion:**

The objective of this meta-analysis was to investigate the influence of external treatment of traditional Chinese medicine on diabetic peripheral vascular disease and to provide more methods for the clinical prevention and treatment of diabetic peripheral vascular disease.

## 1. Introduction

Diabetic peripheral vascular disease (DPVD) is a chronic complication of the large, middle, and small peripheral arteries of the lower limbs in patients with long-term diabetes,^[1]^ including dry skin of the lower limbs, numbness and cooling of limbs, acid distension, resting pain, and intermittent lameness so on.^[2]^ In the later stages of severe cases, dry diabetic gangrene of the affected limb can appear.^[3,4]^ DPVD is a chronic progressive vascular disease that evolves from atherosclerosis, and blood circulation is not smooth due to the thickening of the vascular wall and stenosis of the lumen, eventually leading to ischemia, hypoxia, and even necrosis of the extremities,^[5,6]^DPVD has become an important cause of disability and death in diabetic patients.^[7]^It is predicted that 579 million people will have diabetes in 2030, and the number will increase by 51% (700 million) in 2045.^[8]^

At present, the main treatment for diabetic peripheral vascular disease is blood glucose control, supplemented by anticoagulation, thrombolysis, and vasodilator drugs; drug therapy involves the administration of aspirin, phosphodiesterase inhibitors, prostaglandins, adrenergic receptor inhibitors.^[9]^These drugs have certain limitations, and the therapeutic effect is not idea.^[10]^The external treatment of traditional Chinese medicine (TCM) includes acupuncture, massage, and acupoint application, which has a very good effect on DPVD and is safer.^[11]^

According to ancient TCM records, acupuncture, massage, acupoint application, and other treatments can promote blood circulation and improve nerve function.^[12,13]^ Previous randomized controlled trials (RCTs) have confirmed the effectiveness of external TCM therapy in the treatment of DPVD.^[14]^ However, to date, there have been no systematic reviews or rigorously designed meta-analyses to evaluate TCM external treatment of DPVD. Therefore, our research team plans to conduct a systematic review and meta-analysis through typical TCM external treatment methods such as acupuncture massage and acupoint application to provide real and reliable evidence for the clinical efficacy and safety of DPVD in future studies.

## 2. Methods

### 2.1. Study registration

This protocol adhered to the preferred reporting items for systematic reviews and meta-analyses in 2015.^[15]^

### 2.2. Inclusion criteria

#### 2.2.1. Types of studies.

All RCTs that stated the “randomization” phrase will be included, regardless of allocation concealment or use of blinding, and published or unpublished RCTs without language restriction. Full and peer-reviewed studies should be available.

#### 2.2.2. Types of participants.

This study will employ the diagnostic standards of the Clinical Guideline for DPVD of the American Diabetes Association,^[9]^ and no sex, race, nationality, or comorbidity was limited.

#### 2.2.3. Interventions and comparators.

The experimental group will include all types of external TCM treatments for diabetic peripheral vascular disease, such as acupuncture, tuina, moxibustion, acupoint sticking, and auricular acupuncture. The control group was treated with sham acupuncture, placebo, or conventional medicine, or no treatment. When studies combine 1 of the external TCM treatments with other active therapies, both experimental and control groups are required to use the same active therapy.

#### 2.2.4. Outcome.

The primary outcomes included changes in the bilateral femoral artery, popliteal artery, dorsal artery diameter, blood flow, ankle/brachial index, and dorsal foot temperature; clinical effectiveness rate; and an overall reduction in symptoms (a reduction in diabetes-related symptoms that occur only during the intervention or as a result of the intervention, including peripheral vascular disease).

The secondary outcomes included fasting glucose and glycosylated hemoglobin levels.

### 2.3. Search methods for the identification of studies

The following electronic databases will be searched from the respective dates of database inception to October 20, 2022: Cochrane Library, PubMed, Web of Science, EMBASE, China Science and Technology Journal, China National Knowledge Infrastructure, Wanfang, Chinese Biomedical Literature Database, and other sources.All published English and Chinese RCTs were included.

The retrieval mode used will be a combination of free words and medical subject headings terms, including “diabetic peripheral vascular disease,” “peripheral vascular disease,” “diabetes complications,” “diabetic foot,” “arterial blood flow,” “ankle/ brachial index,” “needling,” “moxibustion,” “Tuina,” “acupoint sticking,” “auricular acupuncture.” The search strategy used PubMed as an example, as shown in Table 1.

**Table 1:** Search strategy for the PubMed database.

Number	Terms
#1	Diabetic peripheral vascular disease (all field)
#2	Peripheral vascular disease (all field)
#3	Diabetes complications (all field)
#4	#1 OR #2–3
#5	Diabetic foot (all field)
#6	Arterial blood flow (all field)
#7	Ankle/brachial index (all field)
#8	#5 OR #6–7
#9	External traditional Chinese medicine (all field)
#10	Needling (all field)
#11	Moxibustion (all field)
#12	Tuina (all field)
#13	Acupoint sticking (all field)
#14	Auricular acupuncture (all field)
#15	#9 OR #10–14
#16	Randomized controlled trial (all field)
#17	Controlled clinical trial (all field)
#18	Randomly (all field)
#19	Randomized (all field)
#20	Random allocation (all field)
#21	Placebo (all field)
#22	Double-blind method (all field)
#23	single-blind method (all field)
#24	Trials (all field)
#25	#16 OR #17–24
#26	#4 And #8 And #15 And #25

### 2.4. Exclusion criteria

The exclusion criteria were as follows: unspecified type or unavailable data for the diabetic peripheral vascular disease population upon request.

### 2.5. Data collection and analysis

#### 2.5.1. Study selection.

**Figure 1.**
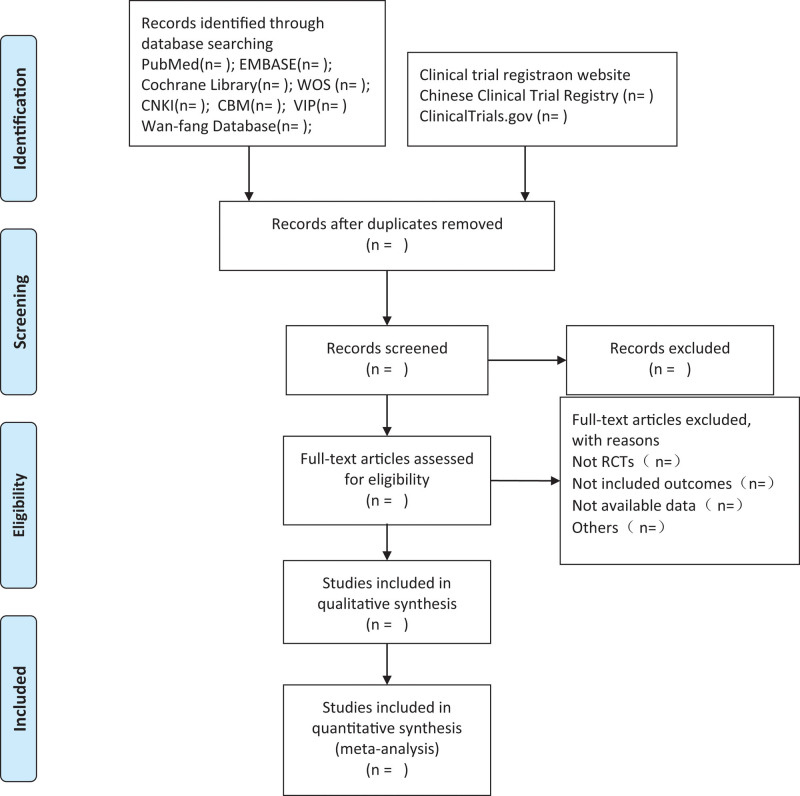
Flow diagram of study selection process.

First, the titles and abstracts of the studies will be independently reviewed by 2 authors (DTW and SL), with preliminary screening of potential leads based on our predetermined eligibility criteria. Second, the full texts of all preliminary selection tests were downloaded to ensure that the eligible tests were met. Unclear or missing information will be supplemented by contacting authors. If there is any disagreement, a third reviewer (ZHY) will participate in the consultation to reach consensus. The preferred reporting items for systematic reviews and meta-analyses (PRISMA) flow chart (Fig. 1) was used to describe the selection process.

#### 2.5.2. Data extraction and management.

The authors (DTW and SL) plan to extract data from the articles selected for inclusion and to resolve differences in opinion through discussion with experts. Data will be recorded on an electronic form, which includes identification information (author, working location, publication date, publication source, etc.), patients (course, sex, age, number), intervention and control (method of intervention, method of control, sample size, and treatment time), and outcomes (treatment efficacy, treatment cycles, side effects, and follow-up).

#### 2.5.3. Assessment of risk of bias in included studies.

The assessment will be conducted by 2 reviewers (DTW and SL) using the risk-of-bias assessment method from Cochrane Handbook 5.0.24.^[16]^ The main content comprised 7 items: generation of random sequence, allocation concealment, blinding of participants and peasonnel, blinding of result evaluators, completion of the outcome data, selective reporting of results, does not include the above 6 kinds of other biases. “High risk,” “low risk,” or “unclear risk” will be used to determine the result above. Disparities will be resolved through discussion and consultation with other authors in our group, and then a judgment will be made based on consensus.

#### 2.5.4. Measures of treatment effects.

Two authors will perform the analysis independently and then crosscheck the treatment effect using the Review Manager software (version 5.3). The risk ratio (RR) with 95% confidence interval (95% CI) was used for dichotomous data. The standard mean difference (SMD) or standard deviation (MD) with 95% confidence interval will present continuous results. Other binary data were converted into RR form.

#### 2.5.5. Dealing with missing data.

To a certain extent, missing data affected the results of this study. Therefore, for studies with missing data, we will contact the corresponding author and request further examination for missing or incomplete data in the record. If relevant data were not available or missing data were not available, we excluded them from the analysis.

#### 2.5.6. Assessment of heterogeneity.

The research will be performed using the Review Manager version 5.3 software. Differences were considered statistically significant at *P* < .05. The *I*^2^ statistic was used to assess the level of heterogeneity in each pairwise comparison. If *I*^2^ was < 50%, the fixed-effects model was used; otherwise, a random-effects model was used. When the results showed substantial or considerable heterogeneity, sensitivity analysis, meta-regression, and subgroup analysis were performed to explore possible sources.

#### 2.5.7. Assessment of reporting bias.

Publication and other reporting biases were assessed using funnel plots. Symmetrical funnel plots showed low bias risk, whereas asymmetric funnel plots showed high bias risk.

#### 2.5.8. Data synthesis.

We also consider whether to conduct a meta-analysis based on clinical research. Meta-analysis was conducted according to the intervention methods, measurement methods, and heterogeneity levels of clinical research. If the clinical and methodological heterogeneity was low, the fixed-effect model of the combined analysis was adopted; when the heterogeneity was at a medium level, the combined analysis adopted a random-effect model.

### 2.6. Sensitivity analysis

We conducted sensitivity analysis to test the robustness of key decisions made during the evaluation process. The central decision node mainly includes the selection of method quality, sample size, and missing data and observes the fluctuation of the results.

### 2.7. Subgroup analysis

If the aforementioned clinical trials lead to significant heterogeneity, we will conduct a subgroup analysis according to the characteristics (interventions, different controls, treatment time, and outcome measurements) of other studies. We tabulated the sources of heterogeneity, and evaluated and explored them.

## 3. Discussion

DPVD is a common chronic disease, serious to the patient’s life and work will bring difficulties, even mental pressure and economic burde.^[17]^External TCM treatment includes many effective techniques, such as acupuncture, moxibustion, massage and acupoint application. The application of external treatment of TCM in the treatment of DPVD has been widely accepted in China, and remarkable results have been achieved.^[18]^Therefore, this study aims to summarize the randomized controlled trial evidence on the treatment of DPVD by various TCM external treatments. To explore the effectiveness, safety and advantages of various TCM external treatment methods in the treatment of DPVD, so as to provide the best treatment methods for clinicians and patients.^[19,20]^

We expect to discover that, compared with lifestyle intervention, external treatment with TCM can be more helpful in preventing and treating DPVD.

## Author contributions

**Conceptualization:** Tingwei Ding, Lei Sheng.

**Data curation:** Haiyu Zhu, Hongyi Guan.

**Formal analysis:** Ying Wang.

**Funding acquisition:** Bailin Song.

**Investigation:** Haoze Guo.

**Methodology:** Yunpeng Bi.

**Supervision:** Bailin Song.

**Validation:** Haoze Guo, Yunpeng Bi.

**Writing – original draft:** Tingwei Ding.

**Writing – review & editing:** Tingwei Ding, Lei Sheng.
